# Volatile Organic Compounds and 16S Metabarcoding in Ice-Stored Red Seabream *Pagrus major*

**DOI:** 10.3390/foods11050666

**Published:** 2022-02-24

**Authors:** Dimitrios A. Anagnostopoulos, Foteini F. Parlapani, Athanasios Mallouchos, Aikaterini Angelidou, Faidra Syropoulou, George Minos, Ioannis S. Boziaris

**Affiliations:** 1 Laboratory of Marketing and Technology of Aquatic Products and Foods, Department of Ichthyology and Aquatic Environment, School of Agricultural Sciences, University of Thessaly, Fytokou Street, 38446 Volos, Greece; anagnostopoulos.dimitriosa@gmail.com (D.A.A.); fwparlap@uth.gr (F.F.P.); kangelidou307@gmail.com (A.A.); faisyropou@uth.gr (F.S.); 2 Laboratory of Food Chemistry and Analysis, Department of Food Science and Human Nutrition, Agricultural University of Athens, 75 Iera Odos, 11855 Athens, Greece; amallouchos@aua.gr; 3 Laboratory of Biology & Histology, Microscopy & Image Analysis, Systematics & Biometry, Department of Nursing, School of Health Sciences, International Hellenic University, 57400 Thessaloniki, Greece; gminos@otenet.gr

**Keywords:** fish, red seabream, spoilage, next generation sequencing, Specific Spoilage Organisms, microbiota, spoilage markers

## Abstract

The profiles of bacterial communities and volatile organic compounds (VOCs) of farmed red seabream (*Pagrus major*) from two batches during ice storage were studied using 16S metabarcoding (culture independent approach) and headspace Solid Phase Micro-Extraction—Gas Chromatography/Mass Spectrometry (SPME-GC/MS) analysis, respectively. Sensory attributes and microbiological parameters were also evaluated. At Day 12 (shelf-life for both batches based on sensory evaluation), using classical microbiological analysis, Total Viable Counts (TVC) were found at the levels of 7–8 log cfu/g, and *Pseudomonas* and/or H_2_S producing bacteria dominated. On the other hand, the culture independent 16S metabarcoding analysis showed that *Psychrobacter* were the most abundant bacteria in fish tissue from batch 1, while *Pseudomonas* and *Psychrobacter* (at lower abundance) were the most abundant in fish from batch 2. Differences were also observed in VOC profiles between the two batches. However, combining the VOC results of the two batches, 15 compounds were found to present a similar trend during fish storage. Of them, 2-methylbutanal, 3-methylbutanal, 3-methyl-1-butanol, ethanol, 2,4 octadiene (2 isomers), ethyl lactate, acetaldehyde and (E)-2-penten-1-ol could be used as potential spoilage markers of red seabream because they increased during storage, mainly due to *Psychrobacter* and/or *Pseudomonas* activity and/or chemical activity (e.g., oxidation). Additionally, VOCs such as propanoic acid, nonanoic acid, decanoic acid, 1-propanol, 3,4-hexanediol and hexane decreased gradually with time, so they could be proposed as freshness markers of red seabream. Such information will be used to develop intelligent approaches for the rapid evaluation of spoilage course in red seabream during ice storage.

## 1. Introduction

Red seabream, *Pagrus major* (Temminck and Schlegel, 1843) is a demersal carnivorous species (Sparidae family) which is widely distributed in the coastal waters of the northwest Pacific, the north-eastern part of the South China Sea (China, Taiwan) northward towards Japan, at depths of 10 to 150 m [1,2]. The intensive farming of the Sparidae species started with the successful artificial breeding of red seabream (*Pagrus major*) larvae in Japan and domestication of seabream (*Sparus aurata*) in the Mediterranean. Over the years, the culture of these two species has evolved into large-scale industries [3]. The fish farming of red seabream in Japan is the oldest among marine fishes; it was the first Sparidae species cultured intensively and it was the first red seabream FAO production derived from Japan due to the production of one tonne in 1958 [3]. Aquaculture production of red seabream increased rapidly during the 1970s when net-cage technology was introduced, and it was only a decade later that production from fish farming was higher than wild fisheries production. Throughout the 1980s and early 1990s, the red sea bream aquaculture sector continued to expand in Japan due to the development of the intensive grow-out production system. During the second half of the 1990s it started to slow down and eventually decreased in the early-middle 2000s [3]. In Japan, red seabream production is the second largest fish culture industry, after amberjacks *Seriola quinqueradiata* and *Seriola dumerili*. The total production of cultured red seabream in Japan has declined from around 80,000 tons in 2004 to 56,861 tons in 2013 as the number of farms has decreased and the costs of feed and of seed from hatcheries have increased. Consequently, the unit price of cultured red sea bream has been rising as production volume has declined [2,4]. It is worth noting that, as of 1999, FAO production statistics of red seabream (*Pagrus major*) are sometimes confused and reported as silver seabream (*Pagrus auratus*) production statistics [3]. In spite of the fact that there is a lack of FAO statistics for this species from China, the red seabream is proven to be an important marine fish species cultured in inshore and offshore cages along the coast of China. Based on a Chinese National report on farmed marine fish species and juvenile production [5], the red sea bream (referred to as *Pagrosomus major*) was the main Sparidae species cultured in the country [3].

Species belonging to the Sparidae family are commercially important, with a strong consumer demand, and red seabream is a high-quality (due to its sensory characteristics e.g., umami flavour, reddish colour), sushi-grade fish with high commercial value and one of the main cultured marine fish species (cultivated in cages) in Japan and Korea [1,2,6]. In Greece, the aquaculture production of red seabream (*Pagrus major*) shows a small upward trend (8% to 9% annually). The annual production was estimated at 1725 mtn in 2018, 1870 mtn in 2019, 2030 mtn in 2020 and 2200 mtn in 2021 [7]. The red seabream is an aquaculture species of high commercial value, widely traded in the Greek market; however, its quality remains unexplored (no available information in the literature).

It is well documented that fresh seafood deteriorates very fast due to the activity of a consortium of bacteria, the so-called Specific Spoilage Organisms (SSOs). In general, the repertoire of SSOs in seafood includes several species belonging to the genera *Pseudomonas* and *Psychrobacter* and, to a lesser extent, *Shewanella* or Lactic acid Bacteria [8]. The dominance of these microorganisms usually differs among seafood depending on a series of factors, such as the applied storage conditions, initial microbiota composition, microbial interaction, type of the product, etc. [9,10]. Therefore, the knowledge of the microbiota present in seafood, how this microbiota is changing under specific storage conditions and what kind of metabolites are produced that finally lead to the sensory rejection, is required in order to maximize the quality of seafood. High-Throughput Sequencing (HTS) techniques and, more specifically, 16S rRNA amplicon-based metataxonomic analysis is currently used to study seafood bacterial communities. This technology has extremely enriched our knowledge regarding the microbiota evolution during storage, as well as the organisms that may be responsible for the deterioration of the sensory attributes of seafood [8,11,12,13,14]. Such information is much more useful when it is combined with the metabolites produced by microorganisms and cause deterioration of seafood freshness. Moreover, other volatile organic compounds which are linked with chemical reactions in chill-stored seafood should also be taken into consideration. Such VOCs, either linked with microbial or chemical activity, have been found to change significantly during storage, indicating a potential role as freshness or spoilage markers of seafood [10]. On the other hand, compounds previously used as spoilage markers of fish, such as TVB-N and TMA, are now considered as unreliable and/or unsuitable to evaluate spoilage course [15,16].

To the best of our knowledge, the microbiota evolution (including the potential SSOs) as well as the VOCs profile (including microbial metabolites that may be linked with the potential SSOs) of red seabream (*Pagrus major*) during ice storage have not been studied. Thus, the aim of the present study was (a) to evaluate sensory, microbiological and VOCs changes, and (b) to assess microbial communities’ composition through 16S metabarcoding in whole red seabream (*Pagrus major*) during ice storage in order to obtain information on microbial spoilage status, and to suggest potential markers for the evaluation of spoilage course in this kind of fish. To achieve this aim, two different batches of fish were taken, because their microbiota and consequently the producing VOCs and shelf-life can be affected by the applied storage conditions and biological variability (e.g., lot-to-lot/batch to batch) [6].

## 2. Materials and Methods

### 2.1. Provision and Storage of Red Seabream

Two batches of whole red seabream (each fish weighed approximately 500 g: 25 Kg each batch) were provided from a Hellenic aquaculture company in July 2019 (Batch 1: 5 July 2019 and Batch 2: 29 July 2019). Red seabream was farmed in the FAO 37, 3.1 geographical area (Aegean Sea). After packaging in insulated boxes with melted ice (one box with fish layers and ice per batch), fish were transferred to the laboratory of Marketing and Technology of Aquatic Products and Foods (University of Thessaly, Volos). The boxes with fish and ice were stored aerobically in an incubator operating at 0 °C for 16 days, while the ice was replaced every 2 days.

Sampling for sensory and microbiological analysis was performed every two days (from D0 to D16), while sampling for 16S metabarcoding and chemical analysis every four days (from D0 to D12—fish shelf-life) for fish from both batches. The samples (four fish for each sampling point) for chemical and 16S metabarcoding analysis were stored at −20 °C until the analysis.

### 2.2. Evaluation of Red Seabream Sensory Rejection

Sensory attributes (skin appearance, presence of slime, texture, odour and colour of flesh, brightness, colour and shape of the eyes) of the whole fish were evaluated by five trained panellists, according to the Multilingual Guide to EU Freshness Grades for Fishery Products [17]. A scale from 5 to 1 was used to score each sensory attribute, with 5, 4, 3 and 2 corresponding to the categories E, A, B and C, respectively, while a score of 1 was attributed to a totally spoiled sample. For general appearance, a score of 3 was considered the score for minimum acceptability, and at any time point, a score below 3 (at least one out of the five panellists scored with 2) was considered the score for rejection (the rejection time point).

### 2.3. Microbiological Analysis

Ten grams (10 g) of tissue from each fish (*n* = 4 fish per batch) was transferred aseptically to stomacher bags with 90 mL MRD (Maximum Recovery Diluent, 0.1% *w*/*v* peptone, 0.85% *w*/*v* NaCl) and homogenized for 2 min using a Stomacher (Bug Mixer, Interscience, London, UK). The spread plate technique (using 0.1 mL of 10-fold serial dilutions) was used for enumeration of the following microorganisms: (a) Total Viable Counts (TVC) on TSA (Tryptone Soy Agar), after incubation at 25 °C for 48–72 h and (b) *Pseudomonas* on cetrimide-fucidin-cephaloridine agar (CFC) incubated at 25 °C for 48 h. The pour plate technique (using 1 mL of 10-fold serial dilutions) was used for the enumeration of (c) H_2_S producing bacteria on Iron Agar Lyngby (IA) by counting only black colonies after incubation at 25 °C for 72 h, (d) Enterobacteriaceae on Violet Red Bile Glucose agar (VRBGA), incubated at 37 °C for 24 h and (e) Lactic Acid Bacteria on De Man, Rogosa, Sharpe agar (MRS) incubated at 25 °C for 72 h. The IA was formulated by its ingredients as follows: peptone 20 g/L, meat extract 3.0 g/L, yeast extract 3.0 g/L, ferric citrate 3.0 g/L, sodium thiosulphate 0.3 g/L, NaCl 5 g/L, L-cysteine 0.6 g/L, agar 14 g/L and pH adjusted at 7.4.

The microbiological media were supplied from LAB M (Lancashire, UK). The results were expressed as mean log cfu/g ± SD (log colony forming unit per g) of four replicates per batch.

### 2.4. 16S Metabarcoding

#### 2.4.1. Samples Preparation and DNA Extraction

Pooled fish flesh of 25 g (*n* = 4 fish per batch) was transferred aseptically to stomacher bags with 225 mL sterile saline solution (0.85% *w*/*v*, 1:10 dilution) and homogenized for 4 min in a Stomacher. Homogenized samples were then transferred to sterile centrifuge tubes and centrifuged (136× *g* for 5 min, 20 °C) to remove any residues. Afterwards, the new supernatants were transferred into new sterile centrifuge tubes followed by a second centrifugation (2067× *g* for 15 min, 20 °C), and the resulting pellet was diluted in 1 mL of sterile deionized H_2_O.

A total of 200 μL of each diluted pellet was used for bacterial DNA extraction using a NucleoSpin Tissue kit (Macherey-Nagel GmbH & Co. KG, Düren, Germany), according to the manufacturer’s instructions. Finally, the concentration and quality of the extracted DNA was evaluated on a nanodrop Quawell UV-Vis Spectrophotometer Q5000 (Quawell Technology, Inc., San Jose, CA, USA).

#### 2.4.2. Library Preparation, Sequencing and Bioinformatic Analysis

The 16S rRNA metabarcoding analysis was applied using the primers 27F (AGRGTTTGATCMTGGCTCAG) and 519Rmodbio (GWATTACCGCGGCKGCTG), as described by Syropoulou et al. [18]. The PCR conditions were as follows: 95 °C for 5 min, followed by 30 cycles of 95 °C for 30 s, 53 °C for 40 s and 72 °C for 1 min, following by a final elongation at 72 °C for 10 min. Finally, all amplicons were mixed in equal concentrations, purified using SPRI beads and sequenced on an MiSeq Illumina platform according to manufacturer’s protocols.

Bioinformatic analysis, including sequences quality filtering, taxonomy classification, rarefaction, and alpha and beta diversity estimation, was applied using the MR DNA ribosomal and functional gene analysis pipeline (www.mrdnalab.com, MR DNA, Shallowater, TX, USA, accessed on 25 December 2021), according to Syropoulou et al. [18].

Finally, raw sequences were deposited in the National Centre for Biotechnology Information (NCBI), under the Bioproject PRJNA789132.

### 2.5. Determination of Volatile Compounds by Headspace SPME-GC/MS

A small portion (approximately 10 g) of fish tissue was cut quickly in small cubes, snap-frozen in liquid nitrogen to quench metabolism and ground for 10–15 s in a pre-cooled A11 analytical mill (IKA, Wilmington, NC, USA) to obtain a fine frozen powder. Aliquots (2 g) of each powdered sample were accurately weighed (±0.01 g) in a porcelain mortar containing 2 g (NH_4_)_2_SO_4_, homogenized for 20 s and transferred into a 20 mL headspace glass vial. Subsequently, headspace SPME combined with GC-MS analysis was carried out as described by Syropoulou et al. [18].

### 2.6. Statistical Analysis

Differences of mean values in microbial counts and sensory score were statistically tested. The data were subjected to Analysis of Variance (ANOVA), followed by Tukey post hoc test using the IBM^®^ SPSS^®^ statistics 19 software (SPSS Inc., Chicago, IL, USA) and a probability level of *p* ≤ 0.05 was considered statistically significant.

Furthermore, to evaluate potential relationships between bacterial diversity-days and VOCs-days, Hierarchical Cluster Analysis (HCA) was performed based on Euclidean distance (similarity measure) and Ward’s linkage (clustering algorithm). Furthermore, a Pearson-based correlogram via univariate analysis was performed to determine potential linkage between bacterial genera and VOCs. Prior to analysis, data were Pareto-scaled, and the results were presented in the form of heatmaps. The analysis was applied using the Metaboanalyst 5.0 platform [19].

The volatiles’ data (peak height) were processed in the Metaboanalyst web platform [20] using univariate (Spearman’s rank correlation test) and multivariate testing (PLS-DA, partial least squares discriminant analysis) [21]. The samples were normalized using an extension of the Probability Quotient Normalization (PQN) method [22], with the samples of storage D0 serving as a pooled reference group. Subsequently, the variables were log10 transformed and auto-scaled prior to statistical analysis.

## 3. Results

### 3.1. Sensory Evaluation of Ice-Stored Red Seabream

At the beginning of fish shelf-life (D0), sensory attributes were scored with 5, for both batches, indicating an excellent quality of the whole fish. The characteristics that started deteriorating first were the skin appearance, colour of eyes and the odour of the fish for both batches.

At D12, the skin was bleached, and its texture was soft, the eyes were slightly concave, had a normal colour but were a bit cloudy, and the odour of the flesh was sour for batch 1 and stale for batch 2. At this time point (D12), the general appearance for fish of both batches was found to be ≥3 (minimum acceptability level), while at D 14 it was found to be below 3 (rejection time point). Therefore, the shelf-life of red seabream was found to be 12 days for fish of both batches.

### 3.2. Microbiological Changes of Ice-Stored Red Seabream

**Figure 1:**
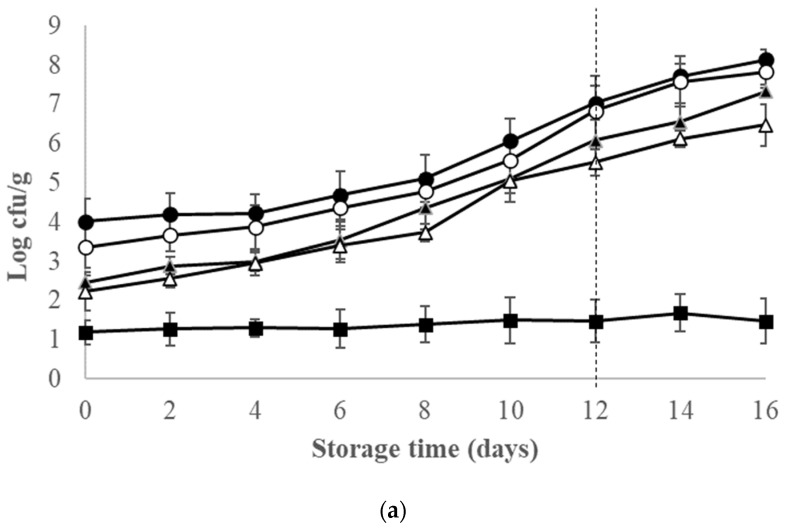

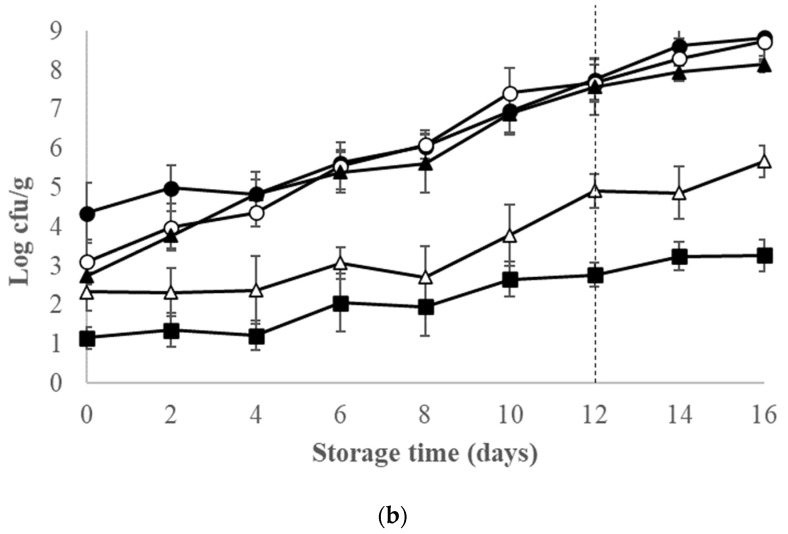
Microbiological changes (TVC (●), *Pseudomonas* (○), H_2_S-producing bacteria (▲), LAB (Δ) and Enterobacteriaceae (■)) of red seabream stored in ice. Each data point and the error bars show the mean and ±st. dev. of 4 replicates (4 fish per batch). The vertical dashed lines indicate the end of shelf-life of the whole red seabream from (**a**) batch 1 and (**b**) batch 2, stored in ice.

At the beginning of fish shelf-life (D0), TVC showed similar population levels for the two batches, 4.01 ± 0.58 (batch 1) and 4.35 ± 0.77 log cfu/g (batch 2). On D 6, 8 and 12, the TVC of batch 2 presented significantly higher counts than those of batch 1 (*p* < 0.05, Figure 1). *Pseudomonas* population was higher than the other microorganisms throughout ice storage. Populations of H_2_S-producing bacteria of the two batches had statistically significant difference (*p* < 0.05) for all days of storage except for D0. Lactic acid bacteria populations were not significantly different, in most cases. Enterobacteriaceae exhibited low counts in both batches, throughout the storage period (Figure 1).

### 3.3. Microbial Communities of Ice-Stored Fish

Table S1 summarizes the results of bioinformatic analysis regarding raw and filtered reads, as well as alpha diversity indices. A total of 147,840 raw reads were obtained, and after quality filtering, 104,679 of them were retained, with an average of 13,085 per sample. Those high-quality sequences were assigned to 234 observed features (range from 15 to 51). Regarding diversity determination, it is crucial to mention that the rarefaction to 5000 sequence depth was more than enough to characterize microbial diversity, because, for instance, the Shannon–Wiener Index curve plot reached a plateau at approximately 500 sequences (Figure S1).

According to metataxonomic analysis, two bacterial phyla, Proteobacteria and Actinobacteria, dominated during whole storage time, while Firmicutes and Bacteroides were found to a lesser extent and in traces, respectively (Figure S2). In all samples, Proteobacteria was the most abundant phylum, while at the end of shelf-life (D12), their relative abundance exceeded more than 90% in both batches. However, the presence of Firmicutes in D8 of the second batch (Pag2_D8) is also noteworthy.

**Figure 2:**
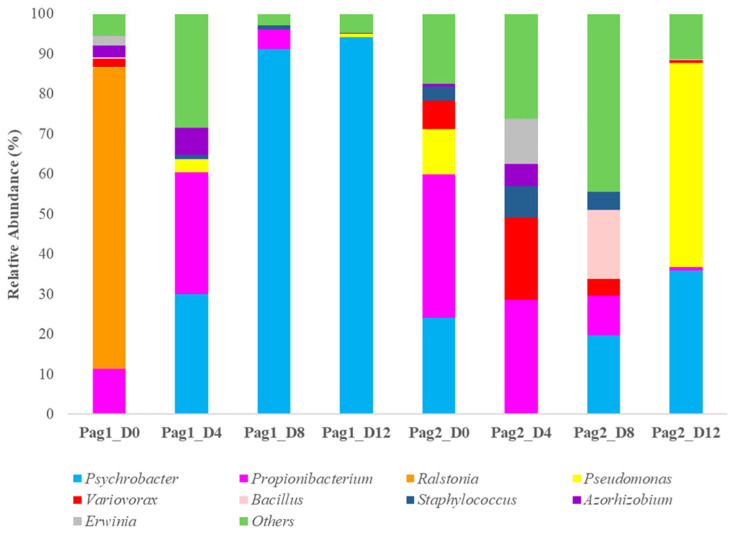
Relative abundance (%) of top-10 bacterial genera of the two batches of red seabream (Pag1, Pag2), revealed through metabarcoding analysis of 16S rRNA gene at intervals of storage time (Days 0, 4, 8 and 12).

At genus level (Figure 2), *Ralstonia* was the most abundant bacteria in the fresh samples of the first batch (Pag1_D0). On the other hand, in the second batch, *Ralstonia* was undetectable, while *Propionibacterium*, which was the most abundant bacteria genera, co-existed with *Psychrobacter* and *Pseudomonas*. Different bacterial profiles were also observed between the two batches in advanced storage time and at the end of shelf-life (D8 and D12). More specifically, in batch 1, the dominance of *Psychrobacter* in D8 and D12 was profound, exhibiting a relative abundance of more than 90%. Conversely, regarding batch 2, several genera, such as *Propionibacterium*, *Bacillus*, *Variovorax* and *Pseudomonas*, coexisted with *Psychrobacter*. It is crucial to mention that, at the end of shelf-life (D12), *Pseudomonas* exhibited higher relative abundance (~51%) compared to *Psychrobacter*, the abundance of which was more limited (~35%).

PCoA revealed a clear separation of samples of both batches at the end of shelf-life (D12) from the rest, even though the sample Pag1_D8 was grouped with them (Figure S3). However, a noteworthy distance between the two batches was observed in fresh samples (D0), as well as in D4 and D8, confirming the differences in microbiota profile between the two batches described above. The principal coordinates explained approximately 89.84% of total variance (factor 1, 2 and 3 explained 40.97%, 30.97% and 17.9%, respectively).

**Figure 3:**
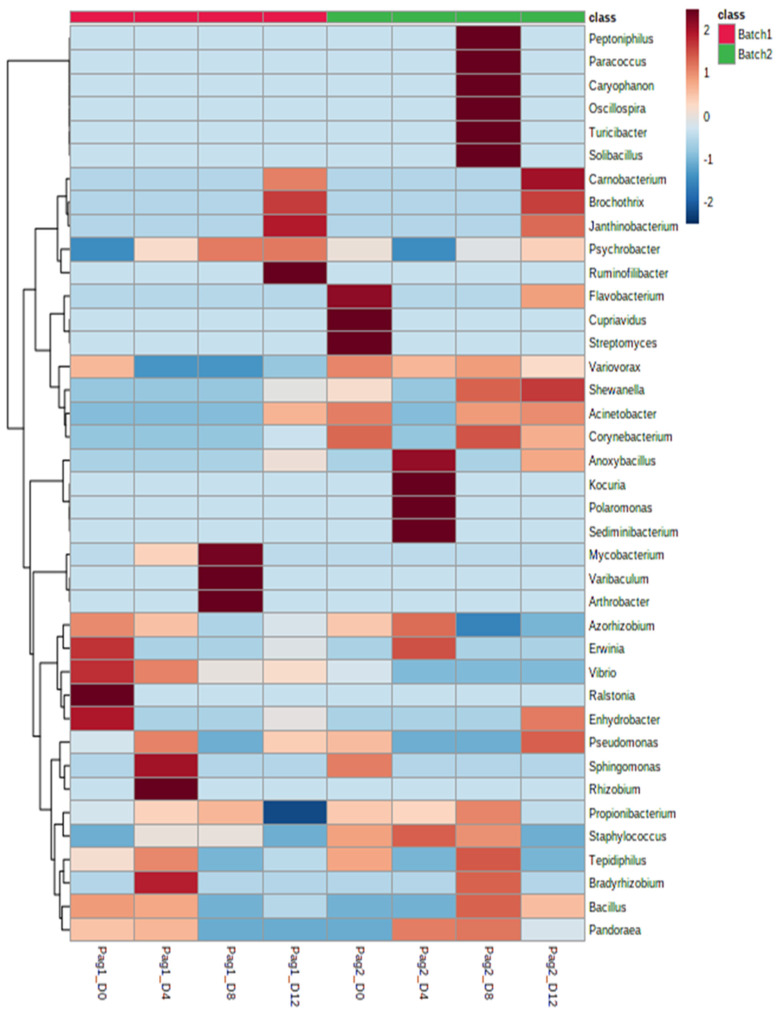
Hierarchically clustered heatmap plot of OTUs (genus level) during storage time per sample.

Based on the clustering heatmap plot (Figure 3), a clear distinction between fresh and spoiled sample profiles was observed, because several genera (i.e., *Azorhizobium*, *Erwinia*, *Vibrio*, *Ralstonia*, *Enzhydrobacter*, *Flavobacterium*, *Cypriavidus*, *Streptomyces*, *Propionibacterium*, *Staphylococcus*, *Pandoraea*) were closely related to the initial stages of storage (D0-D4), while specific bacteria (i.e., *Carnobacterium*, *Brochothrix*, *Janthinobacterium*, *Shewanella*, *Psychrobacter*, *Pseudomonas*) were linked with the end of red seabream’s shelf-life. Notwithstanding that, besides the well-shared bacteria, a noteworthy different profile was observed between the two batches during the whole storage period. For instance, *Flavobacterium* and *Streptomyces* solely existed at the initial stage of storage (D0) of batch 2, while *Shewanella* and *Corynebacterium* were only related to the end of shelf-life of batch 2. Regarding the key spoilage players, *Psychrobacter* was more linked with the end of shelf-life of batch 1, while conversely, *Pseudomonas* was more related to batch 2.

### 3.4. Volatilome during Fish Storage on Ice

Headspace SPME sampling combined with GC-MS analysis was used for the monitoring of volatile profile during fish storage on ice. In total, 104 compounds were identified (Table S2), including mostly aldehydes (18), ketones (11), alcohols (18), acids (10) and hydrocarbons (21 aliphatic and 9 aromatic). A lower number of esters (5), terpenoids (6) and miscellaneous compounds (6) were also detected.

Overall (combining the two batches), the volatilome of red seabream was predominated by alcohols whose content ranged from 47.29% (D0) to 58.23% (D12). 1-Penten-3-ol and ethanol were the most abundant alcohols, accounting together for up to 50% of the total volatile content. The next most abundant chemical classes were aldehydes (ranging from 16.26% at D0 to 22.84% at D12) and ketones (ranging from 6.92% at D0 to 23.43% at D12). Among the carbonyl compounds, acetaldehyde, propanal, hexanal, acetone, 2,3-pentanedione and acetoin presented the highest relative content (>1%). On the contrary, the relative content of the other chemical classes (acids, esters, aromatic hydrocarbons, terpenoids, miscellaneous compounds) did not exceed 3%, except for aliphatic hydrocarbons (ranging from 4.95% to 6.62%).

**Figure 4:**
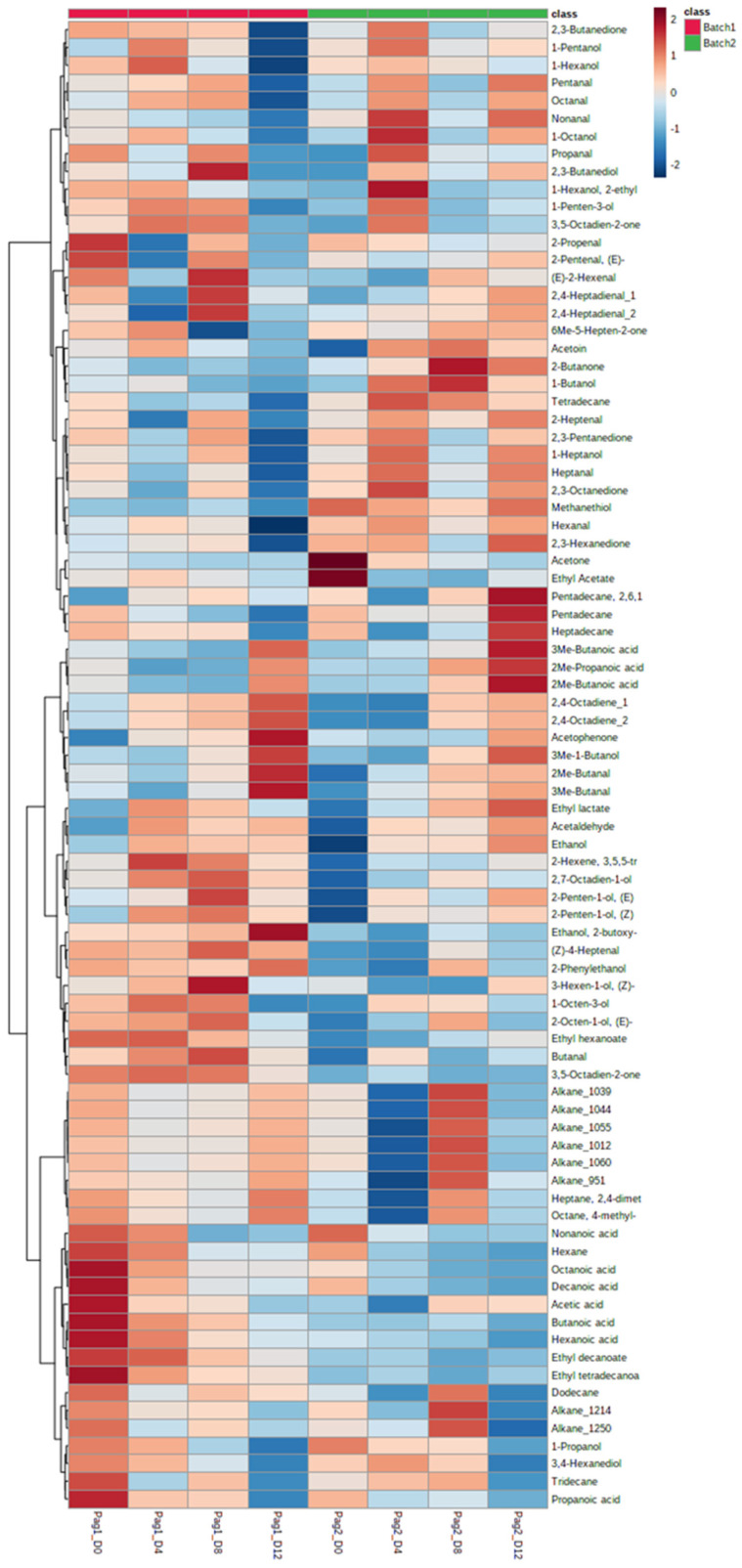
Hierarchically clustered heatmap plot of VOCs during storage time per sample.

Regarding the two batches of fish separately, clustering analysis revealed, besides the common VOCs, a unique profile between batch 1 and 2 at the end of shelf-life (Figure 4). More specifically, a group of volatiles (i.e., acetaldehyde, (E)- and (Z)-2-penten-1-ol, ethanol, 2-methylpropanoic acid, butanoic acid, 2,4-octadiene, acetophenone, butanol and butanal) is well-shared between both batches, while other compounds (i.e., hexanal, heptanal, octanal, pentanal, nonanal, pentadecane, 2,6,10,14-tetramethylpentadecane, heptadecane) are solely linked with batch 2. It is crucial to point out that alkanes, ethanol 2-butoxy, 4-heptanal and 2-phenylethanol are strongly linked with batch 1 (and not with batch 2), at end of shelf-life. Finally, it is worth noting the completely different VOC profiles between the two batches at the initial storage time (D0).

**Figure 5:**
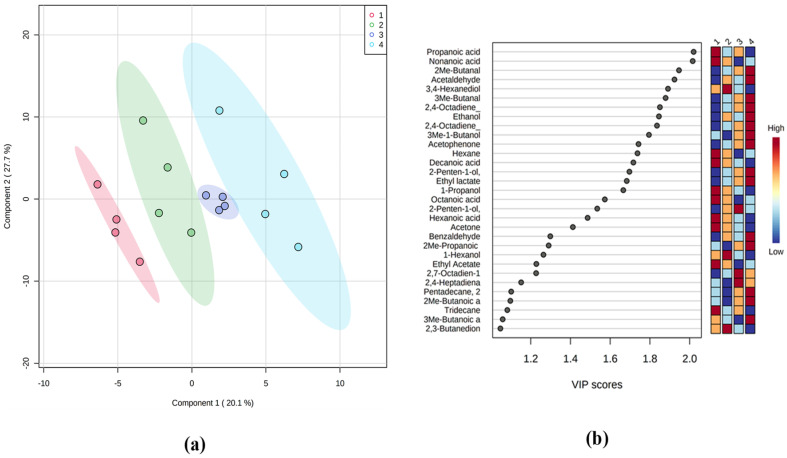
(**a**) PLS-DA modelling of the volatile compounds’ variations during storage of red seabream on ice. The legend indicates days of storage: 1-Day, 0; 2-Day, 4; 3-Day, 8; 4-Day, 12. The percentage of the explained response variance is indicated in parentheses. The shaded ellipses correspond to the 95% confidence regions of each class. (**b**) Volatile compounds with VIP > 1. Colour bars show the normalized content of the volatile compound in the respective class.

To find the volatile compounds showing important variations during the storage period of red seabream in ice, we followed a combination of univariate and multivariate testing. First, PLS-DA was carried out. It is evident that the supervised model (Figure 5a) can discriminate the samples according to storage day. It seems that this separation was described mainly by the 1st principal component. The optimal number of components, as calculated by the leave-one-out cross validation method, was 3. The predictive ability of the model (Q^2^) and coefficient of determination (R^2^) were relatively high (0.97 and 0.84, respectively). The significance of class discrimination was verified by performing a permutation test (empirical *p*-value = 0.036; 36/1000). The variable of importance (VIP) scores were also calculated, and the compounds with VIP > 1 were selected (Figure 5b).

**Figure 6:**
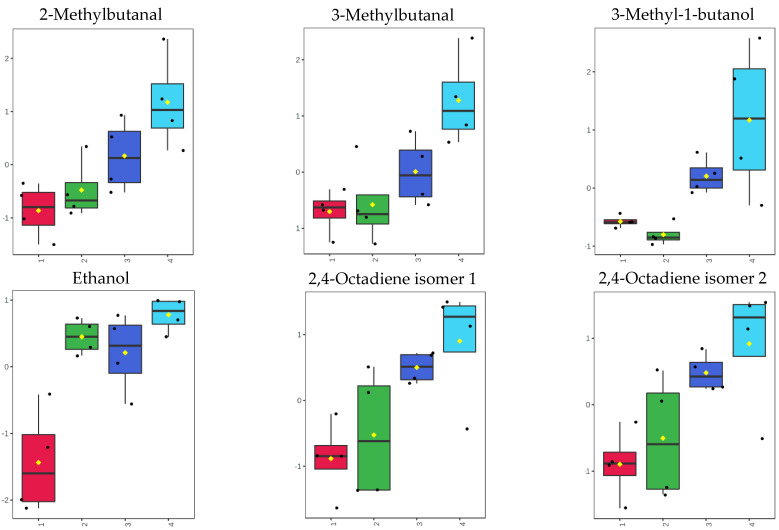

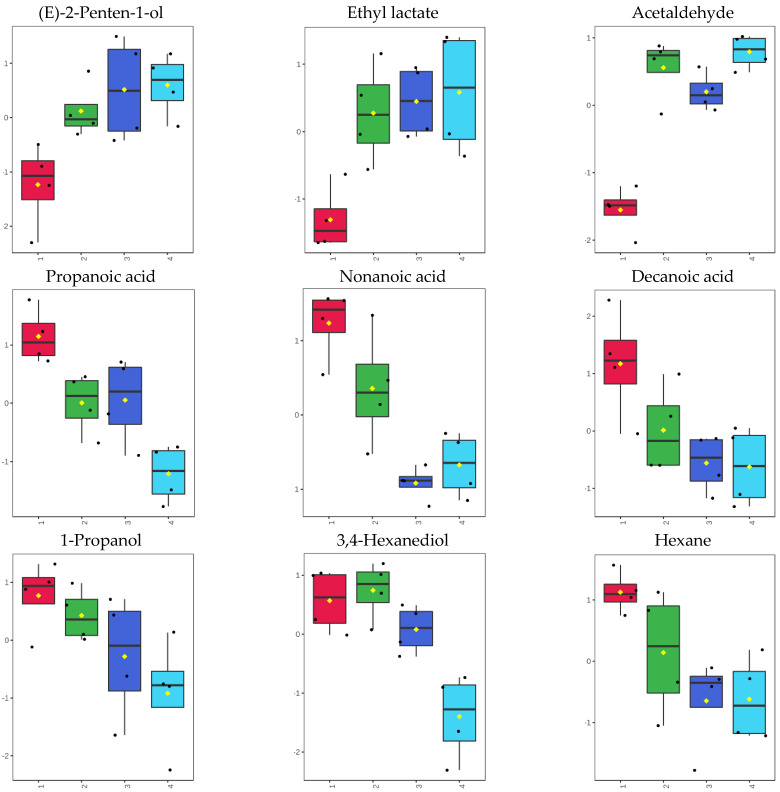
Volatile compounds that either increased or decreased significantly (according to the combined result of PLS-DA and Spearman’s correlation analysis) during storage of red seabream in ice. The numbers (1, 2, 3, 4) on the *x*-axis represent sampling points which are equivalent to the days of storage (D0, D4, D8, D12, respectively) on ice. y-Axis denotes normalized content after log10 transformation and autoscaling.

Second, Spearman’s rank correlation test was conducted using the Pattern Hunter tool of Metaboanalyst to find the volatile compounds which correlated with the increasing days of storage. For each volatile compound, pFDR values (i.e., *p*-values corrected for multiple testing by controlling the false discovery rate at a 5% threshold) were computed and a total of 15 compounds were found significant (*p* < 0.05) with this univariate approach. The combined result of PLS-DA and Spearman’s rank correlation test is summarized in Figure 6, which presents a list of volatile compounds significantly correlated with fish storage in ice. Two groups can be distinguished – the first group includes compounds whose relative content increased during storage, whereas the second group includes compounds showing a decreasing trend.

The correlations between bacterial groups (at genus level) and VOCs are depicted in Figure S4. Among several clusters, it is crucial to note the high correlation of *Psychrobacter* with specific VOCs such as acetophenone, 2,4-octadiene (isomer 1 and 2), 3-methyl-1-butanol, 2-methylbutanal and 3-methylbutanal, while *Pseudomonas* is mainly linked with 2,6,10,14-tetramethylpentadecane. A weaker but not insignificant correlation between *Pseudomonas* and other VOCs (i.e., heptadecane, ethyl acetate, 2-methylpropanoic acid, 2-methylbutanoic acid and 3-methylbutanoic acid) was also observed.

## 4. Discussion

Greece produces and distributes a variety of aquatic products around the world. Among them, fish, e.g., gilt-head seabream and European seabass (highly commercialized fish species) and meagre and red seabream (promising highly commercialized fish species), constitute the most important products of the Hellenic aquaculture, and the majority of them are distributed, traded and consumed as fresh. However, fresh fish spoil rapidly due to bacterial activity. To tackle such commercialization issues, we studied the microbiota evolution as well as the VOCs profile of red seabream during ice storage and obtained information on the microbial spoilage status of this kind of fish, highlighting the potential SSOs and the compounds that may be linked with their activity or other actions (e.g., chemical oxidations).

Red seabream presented similar shelf-life (12 days for both batches based on sensory evaluation) to other ice-stored fish from the Hellenic aquaculture. Shelf-life has been determined from 12 to 16 days for ice-stored gilt-head seabream [23,24] and European seabass [25,26] and from 12 to 15 days for meagre, mainly depending on season of harvesting [18]. Because shelf-life depends on the initial TVC level, microbial composition, microbial activity, microbial interaction, the type of produced VOCs, etc., under the applied storage conditions [9,10], this study showed that the initial TVC level is a crucial parameter that could be improved in fresh red seabream. TVC could be reduced at much more appropriate levels (e.g., 2–3 log cfu/g) than those found herein (4–4.50 log cfu/g) if aquaculturists reinforce Good Hygiene and Manufacturing Practices and Operating Procedures in pre- and post-farm gate, in farming, handling, packaging and distribution. The necessity of such reinforcements has also been obvious through the 16S metabarcoding analysis of the microbial composition of red seabream. Bacteria, e.g., *Ralstonia*, *Propionibacterium*, *Erwinia*, *Staphylococcus*, and *Bacillus*, associated with contamination from various environmental sources, e.g., water, soil, plants, insects, human, were found in fresh fish of both batches. *Ralstonia* was the most abundant genus in the fresh fish of batch 1 (Pag1_D0), while *Propionibacterium* was the most abundant in the fresh fish of batch 2 (Pag2_D0). The aforementioned genera have also been found in other seafood from all around the world [18,25,27,28,29,30,31]. Nevertheless, the detection of these genera increases the possibility of pathogens’ presence in seafood, including the fresh red seabream studied herein, highlighting the need to reinforce the Good Practices and Operating Procedures in the seafood value chain at the global level. For example, *Ralstonia* includes some pathogenic species (e.g., *R. pickettii*, *R. mannitolilytica*, *R. insidiosa*) responsible for nosocomial infection, e.g., infective endocarditis, meningitis, nosocomial pneumonia and bloodstream infection, in immunocompromised patients [32,33]. Of them, *R. pickettii* has been isolated from fish farms in Uganda [34]. *Propionibacterium*, especially the *P. acnes*, constitutes a part of human skin microbiome, sometimes associated with acne vulgaris and other infectious diseases [35]. Such bacteria, also including *Staphylococcus*, can be transferred from infected humans or other contamination sources to seafood and is thus likely to cause infections to food workers and consumers. Of them, only *Staphylococcus*, especially *S. aureus*, is associated with foodborne diseases due to a thermotolerant enterotoxin produced by this microorganism on foods.

Regarding the microbial profile of red seabream during storage, differences were also observed between the two batches. The clear domination of *Psychrobacter* in batch 1 compared to the fish of batch 2, where *Pseudomonas* was the most abundant genus (co-dominating with *Psychrobacter*), is likely to occur due to the different initial microbial composition between the different batches [9,36,37]. Under ice storage conditions, *Psychrobacter* was favoured without its growth being inhibited by the presence of the other microbiota in batch 1, while its fate was different when other microorganisms also existed in fish (in batch 2). This indicates that the initial microbial composition determines the final microbial composition in ice-stored red seabream; the spoilage microbiota, including the SSOs, particularly might depend on the growth requirements of the bacteria and their interaction relationships [10,37]. The latter should be deeply studied in the near future, because it is already known that specific enzymatic activity (e.g., proteolytic) and/or metabolites production of some microorganisms may enhance the growth of others, including SSOs, endowing them with useful ingredients for their biological cycle [38].

*Psychrobacter* has been found to dominate in several seafoods, including finfish and shellfish, from the Hellenic seawaters [11,12,13,18,25,28,39] and from warm and cold waters in other regions [31,40,41,42,43]. *Psychrobacter* and *Pseudomonas* have been recognized as SSOs of seafood due to their ability to produce metabolites that lead to the production of off-odours and the sensory rejection of the products [9,10,44,45]. Herein, according to the clustering analysis, *Psychrobacter* presented a high correlation with VOCs such as acetophenone, octadiene, 3-methyl-1-butanol, 2-methylbutanal and 3-methylbutanal, while *Pseudomonas* presented a high correlation with 2,6,10,14-tetramethylpentadecane and a lower correlation with other VOCs (i.e., heptadecane, ethyl acetate, 2-methylpropanoic acid, 2-methylbutanoic acid and 3-methylbutanoic acid). Moreover, 2-methylbutanal, 3-methylbutanal, 3-methyl-1-butanol, ethanol, 2,4-octadiene, ethyl lactate, acetaldehyde and 2-penten-1-ol were found to increase during storage of red seabream, where *Psychrobacter* and/or *Pseudomonas* dominated. Of them, 3-methyl-1-butanol, 2-methylbutanal, 3-methyl-butanal, 3-methylbutanoic acid, acetaldehyde, ethanol and ethyl acetate, have been proposed as potential spoilage markers of fish because they are associated with the activity of such microorganisms and increase during storage [10,16,18,43,46,47]. Based on the results, the compounds 2-methylbutanal, 3-methylbutanal, 3-methyl-1-butanol, ethanol, 2,4-octadiene, ethyl lactate, acetaldehyde and (E)-2-penten-1-ol are suggested as potential spoilage markers of red seabream stored in ice. On the other hand, compounds such as propanoic acid, nonanoic acid, decanoic acid, 1-propanol, 3,4-hexanediol and hexane decreased gradually with time during storage, due to bacterial or chemical activity [10]. These compounds could be proposed as freshness markers of red seabream. However, more studies have to be performed in order to confirm such results because the quality of this kind of fish has been evaluated for first time herein.

The knowledge of both bacterial composition and VOC profiles during storage allowed us to suggest herein new potential markers as solutions for the rapid evaluation of spoilage course in red seabream, an underexplored fish species from the Hellenic aquaculture with a high potential to enter the world trade dynamically in the coming years. Such information will help fish producers and distributors to know immediately the freshness level of fish so that they can decide at once which fish will be sent in the national, European, US or rest-of-the-world commerce. Therefore, consumers from all over the world will enjoy the delicious and nutritional characteristics of the Hellenic fresh fish. The need of promoting new fish species such as red seabream, with a promising commercial impact on the international commerce, is of great interest, in order to harmonize industry’s interest towards the production of a wide range of fish species that will meet the preferences of different cultures worldwide.

## 5. Conclusions

Shelf-life, microbial and VOCs profile of red seabream were assessed for the first time herein. Fish presented similar shelf-life (D12) between the two batches, but different microbial compositions and VOCs profiles during ice storage. The shelf-life of fresh red seabream might be extended if aquaculturists reinforce GHP and Operating Procedures, in pre- and post-farm gate, thus reducing the initial TVC levels. Such improvements will also reduce the possibility of contamination with bacteria e.g., *Ralstonia*, *Propionibacterium*, *Erwinia*, *Staphylococcus* and *Bacillus*, containing potential pathogens. The presence of different bacteria in red seabream of batch 1 and 2 initially (D0), determined the selection of different SSOs between the two batches (batch 1: exclusively *Psychrobacter*, batch 2: *Pseudomonas* followed by *Psychrobacter*). *Psychrobacter* and *Pseudomonas* were linked with the production of different VOCs, and for this reason the odour was probably sour for the fish of batch 1 and stale for the fish of batch 2, at the end of shelf-life. Despite all these differences, some VOCs, e.g., 2-methylbutanal, 3-methylbutanal, 3-methyl-1-butanol, ethanol, 2,4-octadiene, ethyl lactate, acetaldehyde and (E)-2-penten-1-ol, mainly associated with the activity of such SSOs, were found to increase in both batches, so they are suggested as potential spoilage markers of red seabream stored in ice. Such information will be used to develop various methodologies for the rapid evaluation of spoilage course in red seabream during ice storage to help aquaculturists supply high-quality products in national and international commerce.

## Data Availability

The datasets generated for this study are available on request to the corresponding author.

## Supplementary Materials

The following are available online at https://www.mdpi.com/article/10.3390/foods11050666/s1, Table S1. Number of reads and alpha diversity indices of evaluated samples, Table S2. Relative content (%) of volatile compounds in farmed red seabream during storage on ice. Values represent the mean content of four replicates for each sampling point, Figure S1. Shannon–Wiener rarefaction curves of the two batches of red seabream (Pag1, Pag2), revealed by 16S rRNA metabarcoding analysis at intervals of storage time (Days 0, 4, 8 and 12) through 10 sampling depths, Figure S2. Relative abundance (%) of bacterial phyla of the two batches of red seabream (Pag1, Pag2), revealed through metabarcoding analysis of 16S rRNA gene at intervals of storage time (Days 0, 4, 8 and 12), Figure S3. Principal coordinate plot based on weighted UniFrac distance. Different colour corresponds to different batch/day. Figure S4. Hierarchically clustered correlation heatmap plot of the top OTUs and VOCs.
