# Supplementary material for: Volatile Organic Compounds and 16S Metabarcoding in Ice-Stored Red Seabream Pagrus major
Source: Foods. 2022 Feb 24;11(5):666. doi: 10.3390/foods11050666 (PMC8909714; doi:10.3390/foods11050666)
Supplement: Supplementary file 1 [file foods-11-00666-s001.zip › foods-1557747-supplementary.pdf]

Table S1. Number of reads and alpha diversity indices of evaluated samples.

Sample	Number of Raw Reads	Number of Filtered Reads	Observed Features	Shannon	Faith_pd
Pag1_D0	10,422	7197	51	4.08	32.24
Pag1_D4	11,938	7145	32	3.8	20.63
Pag1_D8	24,744	19,076	16	2.58	1.86
Pag1_D12	21,258	13,440	30	2.61	5.41
Pag2_D0	16,514	13,445	18	3.44	4.83
Pag2_D4	15,118	11,615	15	3.21	4.19
Pag2_D8	19,152	14,541	28	4.37	4
Pag2_D12	28,694	18,220	44	4.58	3.73

Table S2. Relative content (%) of volatile compounds in farmed red seabream during storage on ice. Values represent the mean content of four replicates for each sampling point.

Compound	Retention Index [1]	RID [2]	Storage Period (Days)			
			0	4	8	12
Ethanol	936	A	6.00	19.22	18.43	25.87
1-Propanol	1047	A	0.16	0.10	0.09	0.06
1-Butanol	1163	A	0.15	0.16	0.17	0.12
1-Penten-3-ol	1176	B	34.18	31.03	30.80	25.95
1-Butanol, 3-methyl-	1224	A	0.07	0.05	0.09	0.18
1-Pentanol	1263	A	0.69	0.75	0.62	0.48
(E)-2-Penten-1-ol	1320	B	0.21	0.22	0.28	0.25
(Z)-2-Penten-1-ol	1328	B	2.00	2.92	3.40	2.88
3,4-Hexanediol	1344	C	1.25	1.00	0.88	0.44
1-Hexanol	1360	A	0.38	0.33	0.29	0.22
(Z)-3-Hexen-1-ol	1390	B	0.03	0.03	0.03	0.03

Compound	Retention Index [1]	RID [2]	Storage Period (Days)			
			0	4	8	12
2-Butoxy-ethanol	1407	B	0.13	0.09	0.13	0.18
1-Octen-3-ol	1456	B	1.44	1.30	1.40	1.08
1-Heptanol	1461	B	0.13	0.11	0.11	0.09
2-Ethyl-1-hexanol	1495	A	0.22	0.25	0.17	0.16
1-Octanol	1565	A	0.06	0.07	0.05	0.05
2,3-Butanediol	1584	B	0.08	0.07	0.10	0.07
(E)-2-Octen-1-ol	1622	B	0.07	0.06	0.08	0.05
2-Phenylethanol	1900	A	0.06	0.04	0.05	0.05
Sum of Alcohols			47.29	57.79	57.17	58.23
Acetaldehyde	663	A	2.77	10.61	8.83	12.67
Propanal	786	B	6.08	5.43	5.95	4.43
2-Propenal	841	B	0.06	0.03	0.04	0.03
Butanal	875	B	0.46	0.44	0.47	0.41
2-Methylbutanal	912	B	0.03	0.02	0.04	0.05
3-Methylbutanal	916	B	0.08	0.06	0.09	0.16
Pentanal	973	B	0.61	0.56	0.55	0.49
Hexanal	1086	A	4.05	3.62	3.38	2.75
(E)-2-Pentenal	1134	B	0.13	0.07	0.10	0.08
Heptanal	1188	B	0.25	0.20	0.19	0.17
(E)-2-Hexenal	1222	B	0.11	0.07	0.12	0.08
(Z)-4-Heptenal	1245	B	0.11	0.08	0.13	0.10
Octanal	1290	B	0.26	0.25	0.24	0.20
2-Heptenal	1324	C	0.04	0.03	0.04	0.03
Nonanal	1394	B	0.22	0.24	0.15	0.18
2,4-Heptadienal_isomer_1	1464	B	0.41	0.27	0.46	0.38
2,4-Heptadienal_isomer_2	1492	B	0.43	0.29	0.47	0.36
Benzaldehyde	1520	A	0.18	0.17	0.18	0.24
Sum of Aldehydes			16.26	22.44	21.45	22.84
Acetone	814	A	18.10	3.54	3.09	2.30
2-Butanone	904	B	0.31	0.24	0.79	0.40
2,3-Butanedione	972	A	0.30	0.25	0.25	0.20
2,3-Pentanedione	1064	A	1.43	1.10	1.16	0.91
2,3-Hexanedione	1136	A	0.11	0.09	0.08	0.08
3-Hydroxy-2-butanone (Acetoin)	1289	A	2.22	3.45	3.56	2.27
2,3-Octanedione	1327	B	0.10	0.10	0.08	0.07
6-Methyl-5-hepten-2-one	1340	B	0.03	0.03	0.02	0.02
3,5-Octadien-2-one_isomer_1	1520	B	0.59	0.49	0.51	0.44
3,5-Octadien-2-one_isomer_2	1573	B	0.21	0.21	0.20	0.17
Acetophenone	1650	B	0.03	0.03	0.03	0.05
Sum of Ketones			23.43	9.52	9.77	6.92
Acetic acid	1453	A	0.36	0.18	0.27	0.21
Propanoic acid	1541	A	0.08	0.04	0.05	0.03
2-Methylpropanoic acid	1572	B	0.02	0.01	0.02	0.03
Butanoic acid	1631	A	0.46	0.25	0.27	0.18

Compound	Retention Index [1]	RID [2]	Storage Period (Days)			
			0	4	8	12
3-Methylbutanoic acid	1671	B	0.13	0.09	0.13	0.56
2-Methylbutanoic acid	1673	B	0.04	0.03	0.05	0.11
Hexanoic acid	1847	A	0.78	0.42	0.35	0.27
Octanoic acid	2031	A	0.20	0.09	0.07	0.07
Nonanoic acid	2205	B	0.08	0.04	0.02	0.02
Decanoic acid	2246	A	0.47	0.15	0.11	0.10
Sum of Acids			2.61	1.29	1.33	1.58
Ethyl Acetate	892	A	1.08	0.16	0.12	0.14
Ethyl hexanoate	1239	A	0.03	0.02	0.02	0.02
Ethyl 2-hydroxypropanoate (lactate)	1348	A	0.09	0.43	0.51	0.64
Ethyl decanoate	1644	A	0.04	0.03	0.02	0.02
Ethyl tetradecanoate	2007	A	0.03	0.02	0.02	0.02
Sum of Esters			1.27	0.65	0.70	0.85
Hexane	600	A	0.41	0.17	0.11	0.10
2,4-Dimethylheptane	809	B	0.24	0.10	0.23	0.24
4-Methyloctane	858	B	0.05	0.02	0.04	0.04
2,4-Octadiene_isomer_1	918	C	0.00	0.01	0.02	0.04
2,4-Octadiene_isomer_2	926	C	0.00	0.01	0.01	0.03
Alkane_951	951	-	0.12	0.06	0.16	0.12
Alkane_1012	1012	-	0.18	0.07	0.22	0.14
Alkane_1039	1039	-	0.41	0.16	0.48	0.29
Alkane_1044	1044	-	0.18	0.07	0.20	0.12
Alkane_1055	1055	-	0.06	0.02	0.07	0.05
Alkane_1060	1060	-	0.07	0.03	0.08	0.05
Alkane_1090	1090	C	0.13	0.06	0.14	0.09
Dodecane	1200	A	0.39	0.19	0.37	0.24
Alkane_1214	1214	-	0.07	0.04	0.07	0.03
Alkane_1250	1250	-	0.21	0.12	0.20	0.11
Tridecane	1300	A	0.11	0.07	0.09	0.06
Tetradecane	1400	A	0.09	0.08	0.08	0.06
Alkene_1489	1489	C	2.97	3.07	3.24	2.99
Pentadecane	1500	A	0.07	0.05	0.05	0.05
2,6,10,14-Tetramethylpentadecane	1672	C	0.71	0.53	0.74	0.75
Heptadecane	1700	A	0.06	0.04	0.05	0.05
Sum of Aliphatic Hydrocarbons			6.53	4.95	6.62	5.65
Benzene	933	A	0.06	0.05	0.05	0.04
Toluene	1036	B	0.11	0.07	0.10	0.11
Ethylbenzene	1123	B	0.04	0.02	0.03	0.04
m-Xylene	1137	B	0.04	0.03	0.05	0.05
1-Ethyl-3-methylbenzene	1224	C	0.02	0.01	0.03	0.03
1,3,5-Trimethylbenzene	1242	C	0.02	0.01	0.02	0.02
Styrene	1256	B	0.02	0.01	0.02	0.02
1,2,4-Trimethylbenzene	1278	C	0.04	0.02	0.05	0.05
1-Ethyl-3,5-dimethylbenzene	1322	C	0.01	0.01	0.02	0.02

Compound	Retention Index [1]	RID [2]	Storage Period (Days)			
			0	4	8	12
Sum of Aromatic Hydrocarbons			0.36	0.23	0.39	0.39
a-Pinene	1017	A	0.05	0.02	0.04	0.06
Terpenoid_1048	1048	-	0.04	0.03	0.04	0.07
D-Limonene	1194	A	0.05	0.03	0.04	0.05
p-Cymene	1268	A	0.04	0.02	0.03	0.03
2,6-Dimethyl-7-octen-2-ol	1475	-	0.02	0.03	0.02	0.02
2,7-Octadien-1-ol	1687	C	0.57	0.57	0.74	0.59
Sum of Terpenoids			0.77	0.69	0.91	0.81
Trimethylamine	598	A	0.73	1.91	1.12	2.20
Methanethiol	650	C	0.08	0.05	0.04	0.05
2-Ethylfuran	946	B	0.30	0.20	0.24	0.18
3-Ethylpyridine	1385	B	0.05	0.04	0.04	0.04
2-Ethyl-1H-pyrrole	1621	C	0.10	0.07	0.08	0.07
Butylated hydroxytoluene	1900	B	0.23	0.16	0.13	0.19
Sum of Miscellaneous compounds			1.48	2.43	1.65	2.73

[1] Experimental retention index based on n-alkanes on DB-Wax column. [2] RID - reliability of identification. A-level: agreement of retention index (RI) and mass spectrum (MS) with those of an authentic compound analyzed under identical experimental conditions; B-level: agreement of retention index ($\Delta RI < 20$) and mass spectrum (match > 900); C-level: at least $\Delta RI < 20$ or mass spectrum similarity match > 800.

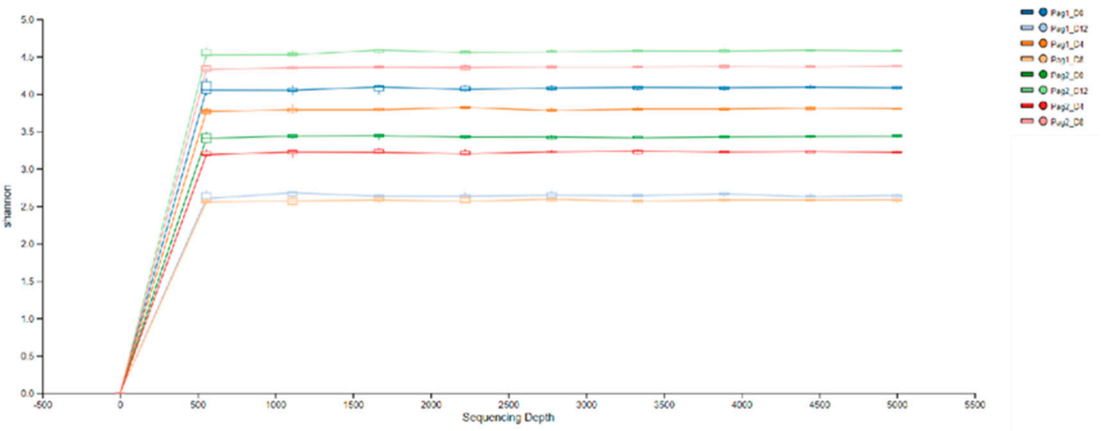

Figure S1. Shannon-Wiener rarefaction curves of the two batches of red seabream (Pag1, Pag2), revealed by 16S rRNA metabarcoding analysis at intervals of storage time (Days 0, 4, 8 and 12) through 10 sampling depths.

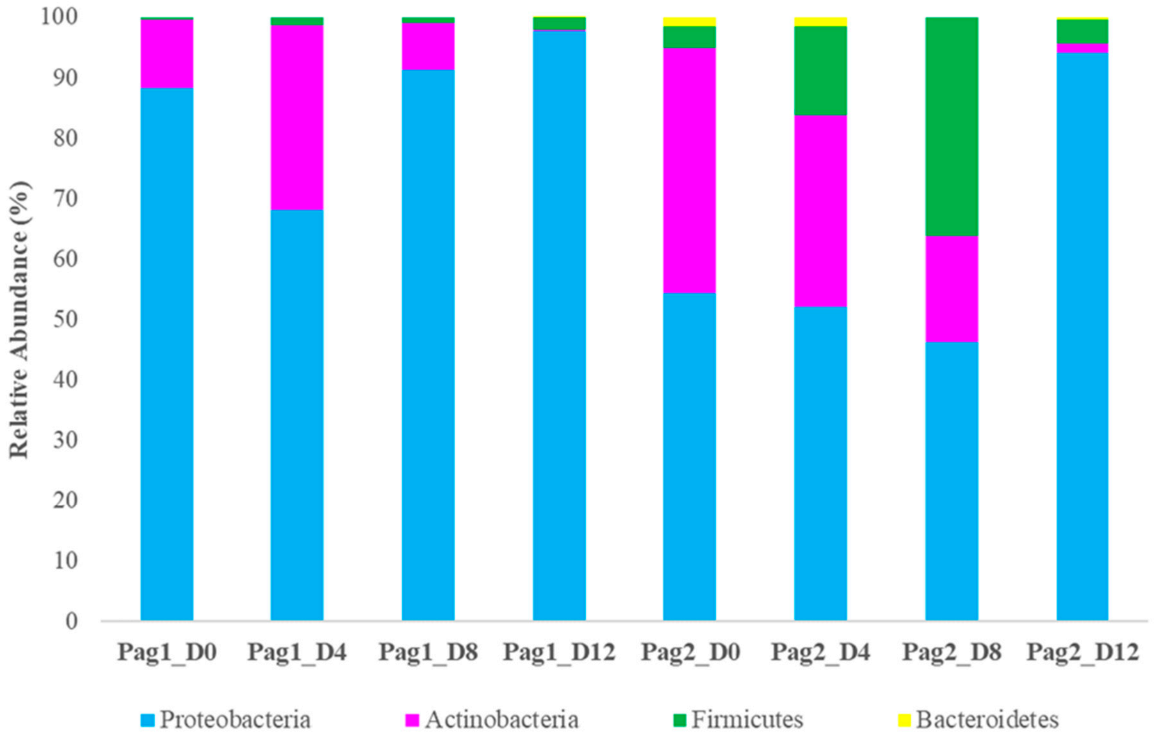

Figure S2. Relative abundance (%) of bacterial phyla of the two batches of red seabream (Pag1, Pag2), revealed through metabarcoding analysis of 16S rRNA gene at intervals of storage time (Days 0, 4, 8 and 12).

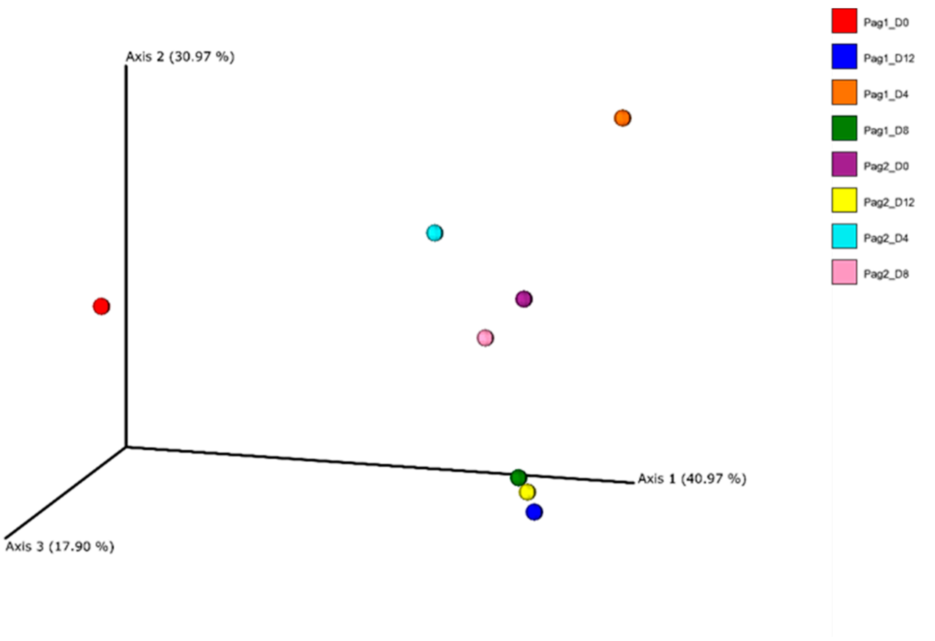

Figure S3. Principal coordinate plot based on weighted UniFrac distance. Different color corresponds to different batch/day.

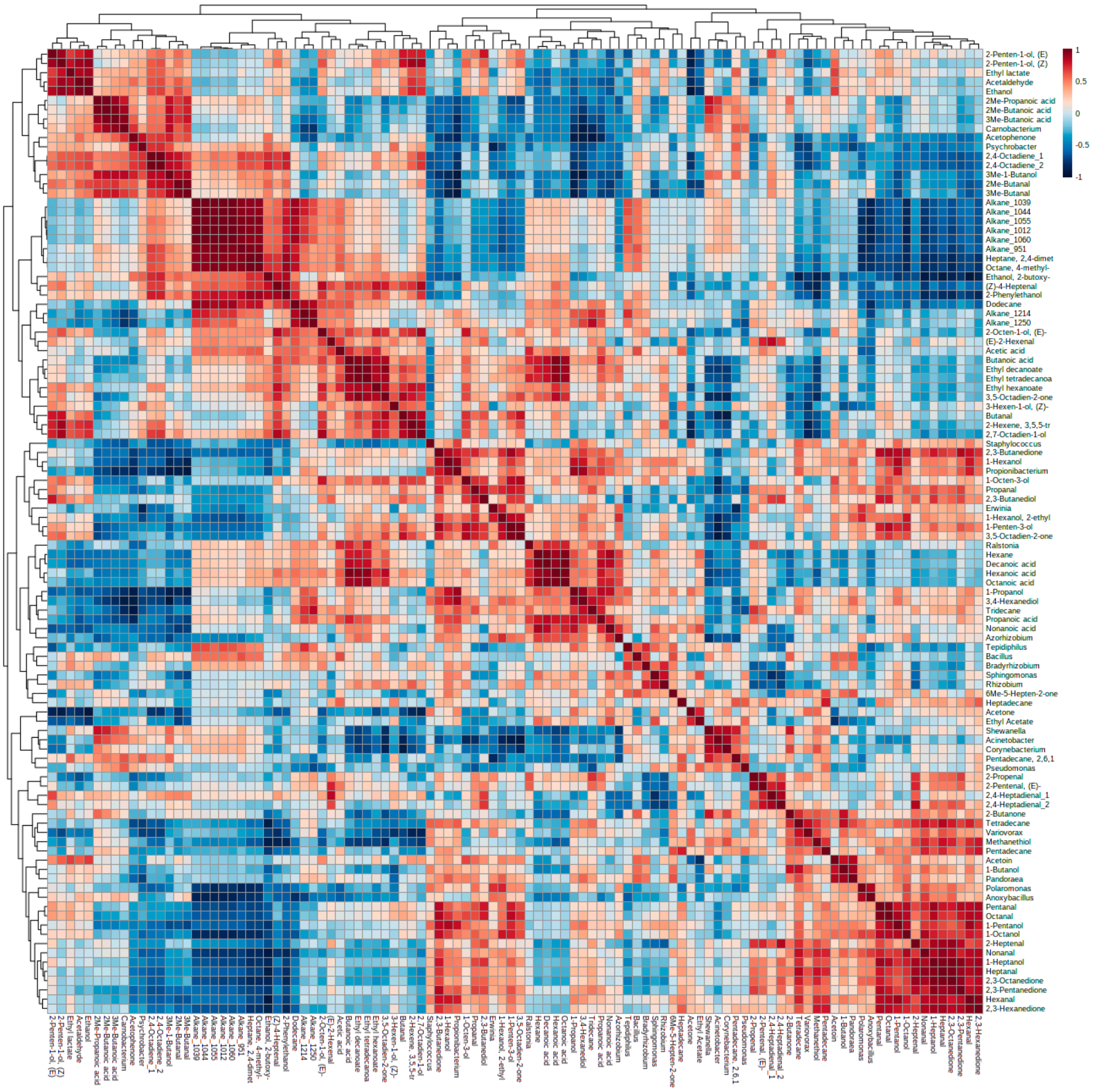

Figure S4. Hierarchically clustered correlation heatmap plot of the top OTUs and VOCs.
